# A Novel Postbiotic From *Lactobacillus rhamnosus* GG With a Beneficial Effect on Intestinal Barrier Function

**DOI:** 10.3389/fmicb.2019.00477

**Published:** 2019-03-14

**Authors:** Jie Gao, Yubin Li, Yu Wan, Tongtong Hu, Liting Liu, Shaojie Yang, Zelong Gong, Qing Zeng, Yi Wei, Weijun Yang, Zhijie Zeng, Xiaolong He, Sheng-He Huang, Hong Cao

**Affiliations:** ^1^Department of Microbiology, Guangdong Provincial Key Laboratory of Tropical Disease Research, School of Public Health, Southern Medical University, Guangzhou, China; ^2^Department of Medical Microbiology and Immunology, Dali University, Dali, China; ^3^Saban Research Institute, Children’s Hospital Los Angeles, University of Southern California, Los Angeles, CA, United States

**Keywords:** probiotic, postbiotic, *Lactobacillus rhamnosus* GG, intestinal barrier function, mucin, tight junction, colitis, bacterial translocation

## Abstract

It has long been known that probiotics can be used to maintain intestinal homeostasis and treat a number of gastrointestinal disorders, but the underlying mechanism has remained obscure. Recently, increasing evidence supports the notion that certain probiotic-derived components, such as bacteriocins, lipoteichoic acids, surface layer protein and secreted protein, have a similar protective role on intestinal barrier function as that of live probiotics. These bioactive components have been named ‘postbiotics’ in the most recent publications. We previously found that the *Lactobacillus rhamnosus* GG (LGG) culture supernatant is able to accelerate the maturation of neonatal intestinal defense and prevent neonatal rats from oral *Escherichia coli* K1 infection. However, the identity of the bioactive constituents has not yet been determined. In this study, using liquid chromatography-tandem mass spectrometry analysis, we identified a novel secreted protein (named HM0539 here) involved in the beneficial effect of LGG culture supernatant. HM0539 was recombinated, purified, and applied for exploring its potential bioactivity *in vitro* and *in vivo*. Our results showed that HM0539 exhibits a potent protective effect on the intestinal barrier, as reflected by enhancing intestinal mucin expression and preventing against lipopolysaccharide (LPS)- or tumor necrosis factor α (TNF-α)-induced intestinal barrier injury, including downregulation of intestinal mucin (MUC2), zonula occludens-1 (ZO-1) and disruption of the intestinal integrity. Using a neonatal rat model of *E. coli* K1 infection via the oral route, we verified that HM0539 is sufficient to promote development of neonatal intestinal defense and prevent against *E. coli* K1 pathogenesis. Moreover, we further extended the role of HM0539 and found it has potential to prevent dextran sulfate sodium (DSS)-induced colitis as well as LPS/D-galactosamine-induced bacterial translocation and liver injury. In conclusion, we identified a novel LGG postbiotic HM0539 which exerts a protective effect on intestinal barrier function. Our findings indicated that HM0539 has potential to become a useful agent for prevention and treatment of intestinal barrier dysfunction- related diseases.

## Introduction

The intestinal barrier is the first defense against harmful microorganisms and antigens invading the gut (Martens et al., 2018). It is a multilayer system mainly consisting of a mucus layer produced by the goblet cells, followed by a monolayer of epithelial cells forming the epithelial tight junction (TJ) (Turner, 2009). The gut immune system and microbiota are also critical components of the intestinal barrier function (König et al., 2016). Disruption of the gut barrier function can result in translocation of pathogens, allergens and luminal toxins through the epithelial layer to lamina propria and then to the mesenteric lymph nodes and can even invade the bloodstream and disseminate to other sterile organs. This process plays a critical role in the pathogenesis of a number of intestinal-related diseases, including irritable bowel syndrome, inflammatory bowel disease, acute liver failure and extra-intestinal infectious diseases (Martín et al., 2016; Xiong et al., 2016; Bron et al., 2017; Vancamelbeke and Vermeire, 2017; Assimakopoulos et al., 2018). Therefore, approaches aimed at reinforcing the intestinal barrier could be of therapeutic interest, in both the prevention and treatment of these pathologies.

One of the effective strategies to reinforce the intestinal barrier is to introduce probiotics, which are defined as ‘live microorganisms that, when administered in adequate amounts, confer a health benefit on the host’ (FAO and WHO, 2001). Growing evidence supports the efficacy of certain probiotic strains in protecting intestinal barrier integrity and its restoration after damage (Zuo et al., 2014; Lopetuso et al., 2015; Marchesi et al., 2016; Bron et al., 2017). For instance, VSL#3, a mixture of lactobacilli and bifidobacteria (*Bifidobacterium infantis*, *Bifidobacterium breve*, *Bifidobacterium longum*, *Lactobacillus plantarum*, *Lactobacillus casei*, *Lactobacillus delbrueckii* ssp. *Bulgaricus*, and *Lactobacillus acidophilus*) can prevent the increase in intestinal permeability in different disease models and also exhibits a beneficial effect on gut barrier function and disease states in clinical studies (Biagioli et al., 2018). *Lactobacillus rhamnosus GG* (LGG), a Gram-positive commensal inhabitant isolated from the gut of a healthy human, is a well-described probiotic strain both in animal models and clinical trials (Segers and Lebeer, 2014). It has shown protective effects on intestinal barrier functions such as immune modulation and maintenance of intestinal integrity. Its intestinal barrier protective effect can be exerted by preventing proinflammatory cytokine-induced deleterious effects on intestinal TJ structure and function (Donato et al., 2010), or increasing intestinal mucin (MUC2) expression to inhibit enteropathogenic *Escherichia coli* adherence (Mack et al., 1999). Since its discovery, a growing body of evidence suggests it has clinical benefits on gastrointestinal disease, for example celiac disease, inflammatory bowel disease, non-alcoholic fatty liver disease and diarrhea (Yoda et al., 2012; Orlando et al., 2014; Ritze et al., 2014; Segers and Lebeer, 2014; Hill et al., 2018). However, despite the well-studied intestinal barrier protection role of probiotics, the molecular mechanisms of probiotic action are complex and remain largely unknown. In addition, problems related to their uncertain bioavailability, infection risk and the possibility of transferring antibiotic resistance gene, hinder probiotics’ use in clinical therapeutics (Doron and Snydman, 2015; Sharma and Im, 2018; Zawistowska-Rojek and Tyski, 2018).

Recently, evidence demonstrates that probiotic-derived effector molecules have potential to exert intestinal protective property in the same way as their parent probiotics (Yan et al., 2007; Mazmanian et al., 2008; Sanchez et al., 2010; Konishi et al., 2016; Quévrain et al., 2016; Lebeer et al., 2018). Identification and characterization of these “postbiotics,” referring to soluble factors (products or metabolic byproducts) secreted by live bacteria or released after bacterial lysis with physiological benefits to the host, have become a novel strategy for maintaining gut health (Tsilingiri and Rescigno, 2012; Cicenia et al., 2014; Suez and Elinav, 2017; Aguilar-Toalá et al., 2018). Our previous study demonstrated that LGG culture supernatant (LCS) promotes development of neonatal intestinal defense and protects the neonatal rats against oral *E. coli* K1 infection (He et al., 2017). However, the identity of bioactive factors in LCS has not yet been determined. Preliminary experiments indicated that proteinase K treatment could inhibit the beneficial effect of LCS. This prompted us to develop a liquid chromatography-tandem mass spectrometry analysis (LC-MS/MS) of the supernatant in order to identify the potential bioactive protein. In this study, we reported a novel secreted protein derived from LGG, named HM0539, with a beneficial effect on intestinal barrier function.

## Materials and Methods

### Ethics Statement

This study was approved by the Medical Ethics Committee of Southern Medical University (Guangzhou, China) and performed according to the guidelines for the protection of animal subjects. All efforts were made to reduce the number of animals used. Eight- to ten-week-old C57BL/6 mice (24–28 g) and timed-pregnant Sprague-Dawley rats were obtained from the Animal Experimental Center of Southern Medical University (Protocol number: L2018018). All animals were pathogen free and were kept in the animal facility under a strict 12 h light/dark cycle. All surgeries were performed under anesthesia with ketamine and lidocaine, and utmost efforts were taken to minimize suffering.

### Bacterial Culture, LCS Preparation and Treatment

LGG culture supernatant was prepared as described previously (He et al., 2017). LGG (ATCC 53103) was kindly provided by Prof. Sheng-He Huang (Children’s Hospital Los Angeles, University of Southern California) and propagated microaerophilicly in De Man, Rogosa, and Sharpe (MRS) broth (Guangdong Huankai Microbial Science and Technology Co. Ltd., Guangzhou, China) at 37°C. LGG was incubated in 5 ml MRS broth at a temperature of 37°C for 18 h under microaerophilic conditions for three generations before the preparation of LCS. Afterward, LGG was streak-inoculated onto the MRS agar and incubated at 37°C for 36 h under microaerophilic conditions. A single colony was picked with a micropipette tip and added to the conical flask with 300 ml MRS broth and incubated at 37°C for 24 h under microaerophilic conditions. Then the culture supernatant was collected and concentrated through an ultrafiltration device (Merck Millipore) with a 3,000-dalton mol wt cutoff. To preliminarily determine the characteristics of the bioactive molecule in the culture supernatant, LCS was pretreated with proteinase K, carbohydrase or DNAse according to following process (Harb et al., 2013): (1) proteinase K treatment: 1 ml LCS was digested with 10 μL proteinase K (52 Unit/mg, Solarbio, Beijing, China) for 10 min at 37°C. Then 20 μL proteinase inhibitor was added. (2) DNAase treatment: 1 ml LCS was digested with 10 μl DNAase (Tiangen Biotech Co., Ltd., Beijing, China) for 25 min at 37°C. Then 10 μl DNAase inactivator was added. (3) β-glucosidase and cellulase treatment: 1 ml LCS was digested with 10 μl β-glucosidase and 10 μl cellulase (Sigma-Aldrich, Germany) for 4 h at 50°C. All the products of digestion were collected and used in mucin-stimulating experiment as described below.

### LC/MS-MS Analysis

LGG culture supernatant was precipitated using trichloroacetic acid and separated by SDS-PAGE. Then the lane of protein sample from LCS was excised and processed for in-gel digestion with sequencing grade trypsin (Promega; Pharmacia Biotech) as previously described (Katayama et al., 2001). Afterward, dried samples were resuspended in 5% acetonitrile and 0.1% formic acid in water for analysis by LC-MS/MS analysis. LC/MS-MS was performed on digested peptides by the ChromXP C18 column (75 μm × 15 cm, C18, 3 μm 120 Å) with spray tip. Data acquisition was performed with a Triple TOF 5600 System (AB SCIEX, United States) fitted with a Nanospray III source (AB SCIEX, United States) and a pulled quartz tip as the emitter (New Objectives, United States).

### Generation and Purification of His-Tagged HM0539 Recombinant Protein

Briefly, LGG genomic DNA was isolated and used as a template for PCR. Considering the transmembrane structure at the N-terminal of HM0539, primers aimed at 51–357 amino sequences were designed for amplification of extracellular protein fragment:

F: 5′-GTAGAATTCGTTAACGCGGCAACGAAAG

R: 5′-CGGGCCTCGAGTTAGTTGATCACTTCAA.

PCR products were purified using a GenElute^TM^ PCR Clean-Up Kit (Sigma-Aldrich), and then digested with *EcoR I* and *Xho I*, followed by ligating to the same restriction enzyme cutting sites of pET-31a(+) (Novagen). The recombinant plasmid was transformed into *E. coli* DH5α (Tiangen Biotech Co., Ltd., Beijing, China). After incubation, the kanamycin resistant bacterial colony was lysed for PCR assay. The expression plasmid producing the HM0539 was confirmed by DNA sequencing and transformed into *E. coli* BL21(DE3) (Tiangen Biotech Co., Ltd., Beijing, China). Recombinant HM0539 protein with a histidine tag was overexpressed via 0.5 mM isopropyl-β-D-thiogalactoside induction at 37°C and purified using Histidine Tagged Protein Purification Kit (Soluble Protein, CW0894, CWBioTech, Beijing, China), according to the manufacturer’s instructions. The potential endotoxin of HM0539 was removed using resin according to the supplier’s instructions (EndotoxinOUT Resin; Sangon Biotech, Shanghai, China).

### Cell Culture and Treatments

Intestinal epithelial cell line Caco-2 was obtained from Shanghai Institute of Cell Biology (Shanghai, China) and grown at 37°C and 5% CO_2_ in Eagle’s Minimum Essential Medium containing 10% heat-inactivated fetal bovine serum (FBS, PAN Biotech, Aidenbach, Germany), penicillin (100 units/ml, HyClone, United States) and streptomycin (100 μg/ml, HyClone, United States). To allow Caco-2 to fully differentiate, cells were grown for a minimum of 12 days (Wang et al., 2011). To detect the beneficial effect of HM0539, we seeded Caco-2 into 24-well plates for periodic acid Schiff (PAS) staining, immunofluorescence assay and transepithelial electrical resistance (TEER) determination; and 6-well plates for western blot analysis. Differentiated Caco-2 incubated with different concentrations of bovine serum albumin (control, 50 ng/ml) or HM0539 (50 ng/ml) for 12 h. Afterward, Caco-2 monolayers were stimulated with tumor necrosis factor-α (TNF-α, 10 ng/ml, Sigma-Aldrich) or lipopolysaccharide (LPS, 1 μg/ml, Sigma-Aldrich). After 6 h stimulation, Caco-2 monolayers were applied for PAS staining as described below. The expressions of ZO-1, occludin and MUC2 were detected using western blot or immunofluorescence staining.

### PAS Assay

For the colorimetric method, treated Caco-2 monolayers were harvested, lysed in radioimmunoprecipitation assay lysis buffer and treated as described previously (Wang et al., 2014). Cellular lysis was collected and centrifuged at 12,000 *g* for 10 min at 4°C. Soluble ingredients in the supernatant were harvested and protein content was detected by bicinchoninic acid assay kit (Beyotime Institute of Biotechnology, China). Afterward, 50 μg protein was mixed with 0.1% periodic acid in a 96-well microtiter plate and incubated for 2 h at 37°C, followed by adding equal volume of Schiff reagent for 30 min in darkness at room temperature. The production of mucin was monitored at 550 nm using a microplate reader. All samples were analyzed in triplicate.

To detect the mucin covering the Caco-2 monolayers, treated cells were fixed with 4% paraformaldehyde for 10 min at room temperature. This was followed by washing with distilled water several times and staining using a PAS staining kit (Solarbio Science & Technology Co., Ltd., Beijing, China) according to the manufacturer’s instructions. Afterward, all Caco-2 monolayers were counterstained with hematoxylin and visualized using light microscopy.

### Western Blotting for Protein Expression Measurement

Cells were lysed directly by 200 μl of 1 × SDS-PAGE loading buffer. Cell lysates were separated using SDS-PAGE and transferred onto polyvinylidene difluoride membranes (pore size, 0.45 μm, Millipore, United States). Then, the membranes were blocked with 5% (wt/vol) non-fat milk in TBST buffer [50 mM Tris/HCl, pH 7.4–7.6, 150 mM NaCl and 0.1% (vol/vol) Tween-20] for 1 h, followed by incubation in primary antibody diluted in 5% non-fat milk in TBS-Tween-20 (TBST) for 12 h at 4°C. Afterward, the membranes were washed and probed with the corresponding horseradish peroxidase (HRP)-conjugated secondary antibody diluted in 5% non-fat milk-TBST for 1 h. The expression of antigen was visualized using enhanced chemiluminescence reagent (Pierce Biotechnology, Rockford, IL, United States). The primary antibodies used are as follows: rabbit anti-MUC2 (1:1000), rabbit anti-ZO-1 (1:2000), rabbit anti-occludin (1:1000) and rabbit anti-β-actin (1:10000) (Proteintech Group, Chicago, IL, United States).

### Measurement of TEER

Caco-2 cells were seeded in 24-well Transwell inserts (3 μm pore size, 6.5 mm diameter, Corning Costar Corp., United States) and cultured for 19–21 days. The culture medium was replaced every 2 days. The integrity of Caco-2 monolayers was evaluated by measuring the TEER (EVOMAX, World Precision Instruments, United States). To investigate the protective effect of HM0539 against TNF-α or LPS-induced intestinal integrity disruption, Caco-2 monolayers were pretreated with 50 ng/ml of HM0539 for 12 h, which was followed by adding TNF-α (10 ng/ml) or LPS (1 μg/ml). TEER values were detected at 0, 1, 2, 3, 4, and 5 h post-TNF-α or LPS treatment. The experiments were performed in triplicate.

### Pectin/Zein Hydrogel Drug Delivery System

A pectin/zein beads delivery system was used to deliver HM0539 to the colon as they protect HM0539 from protease attack. The pectin/zein beads were prepared as described previously (Shen et al., 2018). First, 1% (w/v) zein (Sigma-Aldrich, United States) was dissolved in 85% ethyl alcohol solution (adding 0.5% CaCl_2_, w/v) to prepare the zein solution. Meanwhile, His-tagged HM0539 was added to 6% pectin solution (w/v, dissolve in distilled water) to prepare the pectin/HM0539 solution. Afterward, at room temperature, the pectin/HM0539 solution was inhaled into a syringe with a 23 G needle and dropped (approximately 50 μl/drop) into the zein solution gently. The beads were collected after becoming solid in zein solution, and washed using distilled water several times, air dried at room temperature and stored at 4°C. Pectin/zein beads without HM0539 were prepared simultaneously as controls. According to a previous study (Yan et al., 2011), the beads with an average size of 2 mm contained 5 μg protein.

### Model of *E. coli* K1 Infection via the Oral Route

*Escherichia coli* K1 infection via the oral route in neonatal rats was induced as described in our previous study (He et al., 2017). Twelve neonatal rats from 2–3 L were pooled and randomly divided into two groups (*n* = 6). To investigate the effect of HM0539 on the maturation of neonatal intestinal defense, neonatal rats were gavage fed with pectin/zein control or pectin/zein beads containing HM0539 once a day for 3 days without *E. coli* K1 infection. All rats were sacrificed after 3 days of treatment. The expression of intestinal epithelial cell proliferation marker Ki67 and differentiation marker MUC2, as well as mucin and ZO-1 were detected using immunohistochemical analysis and PAS assay. To investigate whether HM0539 could enhance the resistance of neonatal rats to oral *E. coli* K1 infection, animals were gavage fed with pectin/zein control or pectin/zein beads containing HM0539 once a day for 3 days. Afterward, all rats were gavaged with *E. coli* K1 (100 μl, 5 × 10^9^ colony forming unit, CFU). Sixty hours after *E. coli* K1 treatment, all the rats were anesthetized using ketamine and lidocaine. Blood and cerebrospinal fluid (CSF) samples were harvested aseptically and plated onto brain heart infusion agar with rifampin (100 μg/ml) for bacteria counting. To evaluate the intestinal permeability, rats received fluorescein isothiocyanate (FITC)-dextran (Sigma-Aldrich, MW 4000, 60 mg/kg body weight) orally 4 h before sacrifice. Plasma was harvested and FITC fluorescence intensity was measured using a fluorescence spectrophotometer (excitation wavelength = 485 nm, emission wavelength = 535 nm).

### Dextran Sulfate Sodium (DSS)-Colitis Model and Treatment

Twenty-four C57BL/6 mice (8–10 weeks old, 24–28 g) were randomly allocated into three groups (*n* = 8): the control group, the DSS-colitis group, and the HM0539 + DSS-colitis group. In day 1, the control and DSS-colitis groups were gavage fed with pectin/zein control once a day for 9 days, whereas the HM0539 + DSS groups were gavage fed with pectin/zein contain HM0539. From days 3 to 9, the drinking water of DSS-colitis group and HM0539 + DSS-colitis group was changed with 3% DSS (w/v, 40 kDa; Aladdin, Shanghai, China) in water, whereas the control group was administered with the normal drinking water. All the mice were then given normal drinking water for 2 days. During DSS treatment, body weight change was recorded daily. On day 12, all mice were sacrificed, their colons were excised and length was measured. Colon tissues were homogenized and lysed for western blot analysis for detecting the expression of MUC2 and ZO-1. Some colon tissues were fixed with formalin at room temperature and embedded for hematoxylin-eosin (HE) staining and immunohistochemical analysis.

### LPS-D-Galactosamine (LPS/GalN) Model and Treatment

Twenty-one C57BL/6 mice (8–10 weeks old, 24–28 g) were randomly divided into three groups (*n* = 7) with different treatments as follows: control group, LPS/GalN group and HM0539 + LPS/GalN group. From days 1 to 7, both control and LPS/GalN group were gavage fed with pectin/zein control once a day for 7 days, whereas the HM0539 + LPS/GalN group was gavage fed with pectin/zein containing HM0539. On day 8, all the mice except the control group were intraperitoneally injected with LPS (40 μg/kg, from *E. coli* O55:B5, Sigma-Aldrich, United States) and D-galactosamine (360 mg/kg, Aladdin, Shanghai, China), whereas the control group was intraperitoneally injected with an equal volume of saline. To examine bacteria translocation across the intestinal barrier, all mice were sacrificed 24 h later, their liver and mesenteric lymph node (MLN) were harvested aseptically and homogenized to plate on brain heart infusion agar. All plates were incubated anaerobically for 48 h at 37°C. To evaluate the intestinal permeability, mice orally received FITC-dextran (Sigma-Aldrich, MW 4000, 60 mg/kg body weight) 4 h before sacrifice. FITC levels in blood were measured as described above.

### Histopathology and Immunohistochemistry

Fresh liver or colon tissue was fixed in 10% formalin for 2 days and then processed for sectioning and staining by standard histological methods. Sections from the liver and small intestine were stained with HE and evaluated under light microscopy.

For the immunohistochemical staining, paraffin sections were deparaffinized and rehydrated following standard protocols. To retrieve antigen, sections were boiled with 0.01 M citrate buffer (pH 6.0) for 30 min. Afterward, sections were washed and immersed in 3% hydrogen peroxide/methanol for 30 min at room temperature. Then, the sections were blocked with 1% bovine serum albumin and incubated with rabbit anti-MUC2 (1:200) or rabbit anti-ZO-1 (1:200, Proteintech Group, Chicago, IL, United States). The sections were then washed and incubated with the appropriate secondary antibodies, washed and visualized using DAB (3,3=-diaminobenzidine) with hematoxylin counterstain. The optical density of the immunostained areas were quantified using ImageJ software (United States National Institutes of Health).

### Liver Function Tests

Blood samples were extracted and centrifuged after decapitation at 3,000 *g* for 10 min to harvest the serum. Alanine aminotransferase (ALT) and aspartate aminotransferase (AST) levels in the serum were quantified using ALT assay kit and AST kit (Nanjing Jiancheng Bioengineering Institute, Jiangsu, China) according to the supplier’s instructions. The data were obtained using a microtiter plate reader set at 510 nm. All samples were analyzed in triplicate.

### Statistical Analysis

Data are presented as the mean ± standard error (SEM). Analysis between two groups was performed by unpaired two-tailed Student’s *t*-test. Analysis involving more than two groups was examined by two-tailed, one-way analysis of variance (ANOVA) with multiple comparison *post hoc* analysis. All statistical analyses were carried out using the GraphPad Prism software version 7.0. *P* < 0.05 was defined as statistically significant.

## Results

### Characterization of Bioactive Factors From LCS

**FIGURE 1:**
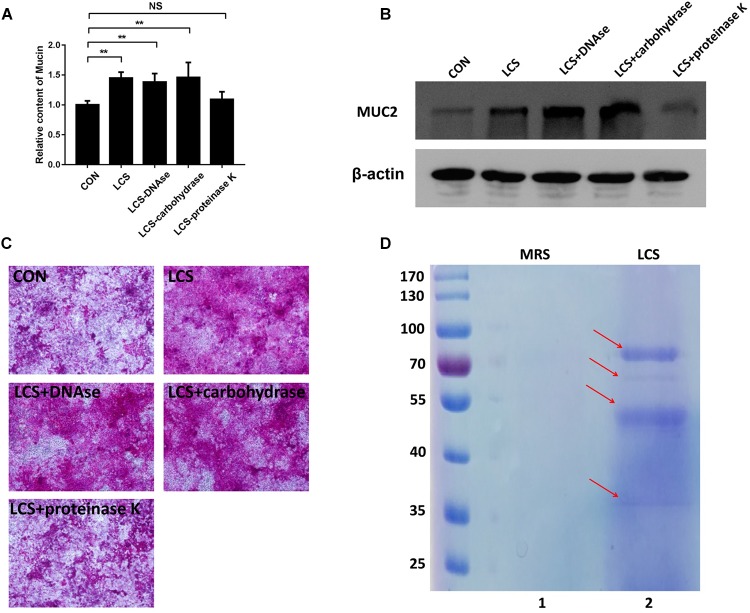
Characterization of soluble protein detected from LCS. LCS was pretreated with proteinase K, DNAse or carbohydrase, then the digested products were applied to treat Caco-2 monolayers, the soluble mucin **(A)** and mucin-covered Caco-2 surface (**C**, upper panel) were detected using PAS assay, expression of MUC2 (**B**, middle and lower panels) was evaluated by immunoblot, β-actin was used as loading control. **(D)** LCS was precipitated and separated by SDS-PAGE (Lane 2). Data are given as means ± SEM; ^∗∗^*P* < 0.01; NS, no statistical significance.

To obtain first insights into the nature of the bioactive factor, LCS was pretreated with protease K, DNase or carbohydrate-digesting enzymes, followed by evaluating its mucin-enhancing ability using PAS assay. We found that only protease K-treated LCS exhibited a significant decrease in mucin production (Figure 1A,C), implying that the bioactive metabolites of LGG may be a kind of protein. A similar result was obtained by western blot analysis of MUC2 (Figure 1B), confirming the finding described above.

The beneficial activity of LCS is sensitive to protease K treatment leading us to analyze the protein composition of LCS by means of SDS-PAGE and coomassie blue staining. Four visible bands had molecular masses of approximately 80, 60, 50, and 37 KDa were observed (Figure 1D). Afterward, the gel was excised and digested to analyze the protein composition by means of LC-MS/MS. The result showed that a total of 58 proteins were identified, and ten of the most abundant were listed in Table 1. As p40 and p75 are the most abundant proteins secreted by LGG reported previously by Yan et al. (2007), we wonder whether these two proteins exist in our LCS. However, we only found p75, the cell wall-associated hydrolase (accession no. gi| 199589812). As previous studies demonstrated that p75 is not the major bioactive factor secreted by LGG (Yan and Polk, 2012), we thus focused on the hypothetical protein HMPREF0539_2242 (abbreviate to HM0539 in this study), the most abundant protein identified in LCS (Tables 1, 2). In searching HM0539 using the NCBI protein data bank, we found it is a putative protein with 37.3 KDa, calculated isoelectric point = 9.22. A BLAST search showed that the amino acid sequence of HM0539 was 92% similar to a hydrolase from *L. rhamnosus* (Table 2 and Supplementary Figure S1A). The sequence alignment of HM0539 to its other LGG counterparts (p40 and p75) revealed low sequence identity with 13.7 and 18.9%, respectively (Supplementary Figure S1A). Full-length coding DNA of the HM0539 protein was shown in the Supplementary Table S1. The information about the genomic locus that harbors HM0539 is shown in Supplementary Figure S2. A genomic BLAST search of sequences available in the GenBank database^1^ revealed that genes encoding HM0539 homologs spread in the genomes of a variety of *L. rhamnosus* strains and *L. casei*. Amino acid comparison results of these best matched homologous proteins to HM0539 are shown in Supplementary Table S2 (using online Sequence Manipulation Suite software) and Supplementary Figure S3 (aligned using DNAMAN program). Bioinformatic analysis using SignalP-4.1 and TMHMM server 2.0 showed that HM0539 contained a signal peptide (1–53 aa) and transmembrane section (29–46 aa), respectively (Supplementary Figures S1B,C). Finally, to further explore the bioactivity of HM0539, we recombined and purified it using the His-tag/Ni-NTA system (Supplementary Figure S1D).

**Table 1:** The ten most abundant proteins detected from LCS using LC-MS/MS analysis.

No.	Accession number	Score	Mass	Sequences	Protein
**(1)**	**gi| 229313674**	**2617**	**37309**	**15 (12)**	**Hypothetical protein HMPREF0539_2242 [*Lactobacillus rhamnosus* LMS2-1]**
(2)	gi| 199589812	1642	49766	10 (8)	Cell wall-associated hydrolase [*Lactobacillus rhamnosus* HN001]
(3)	gi| 1034335721	805	41687	18 (15)	FliK family flagellar hook-length control protein [*Lactobacillus rhamnosus*]
(4)	gi| 229315418	698	45175	15 (13)	Trypsin [*Lactobacillus rhamnosus* LMS2-1]
(5)	gi| 668899397	320	40899	19 (6)	Hypothetical protein LRK_14390 [*Lactobacillus rhamnosus* K32]
(6)	gi| 199591216	313	23257	8 (5)	Cell wall-associated hydrolase [*Lactobacillus rhamnosus* HN001]
(7)	gi| 112022814	303	36929	9 (8)	Sequence 65 from patent US 7052896
(8)	gi| 257147364	293	35881	9 (5)	Pilus specific protein, major backbone protein [*Lactobacillus rhamnosus* GG]
(9)	gi| 257147366	278	96790	10 (8)	Pilus specific protein [*Lactobacillus rhamnosus* GG]
(10)	gi| 199590823	243	21206	6 (5)	Single-stranded DNA-binding protein [*Lactobacillus rhamnosus* HN001]


**Table 2:** Internal peptide sequences of HM0539 detected by LC/MS/MS analysis.

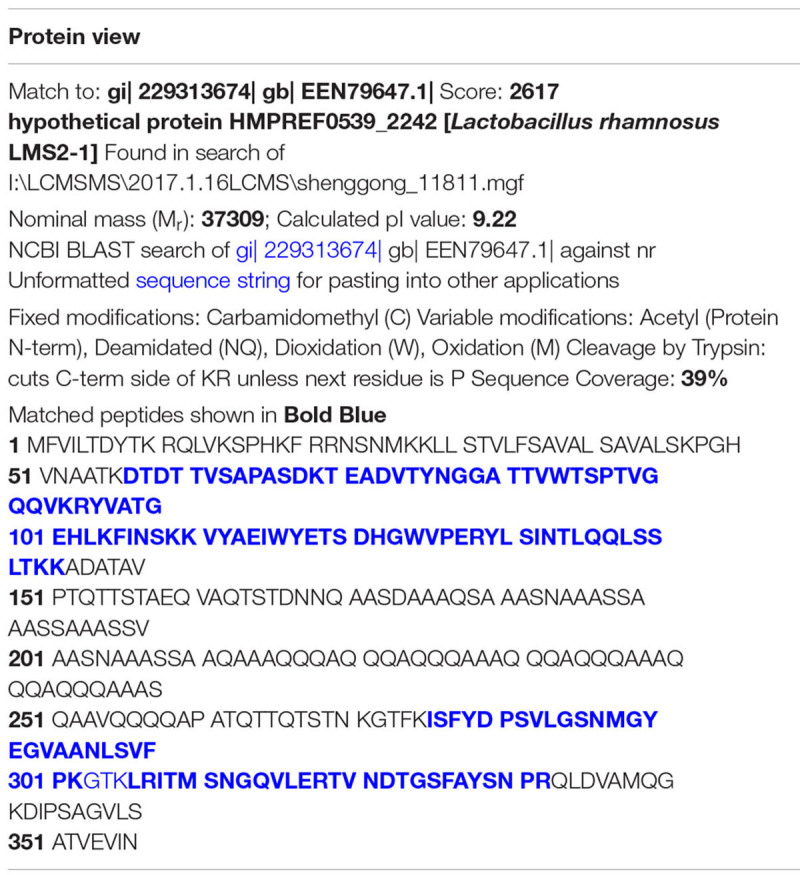

*Bold blue: Internal sequence detected by LC-MS/MS analysis.*.

### Treatment With HM0539 Protects Against LPS- and TNF-α-Induced Intestinal Barrier Injury *in vitro*

**FIGURE 2:**
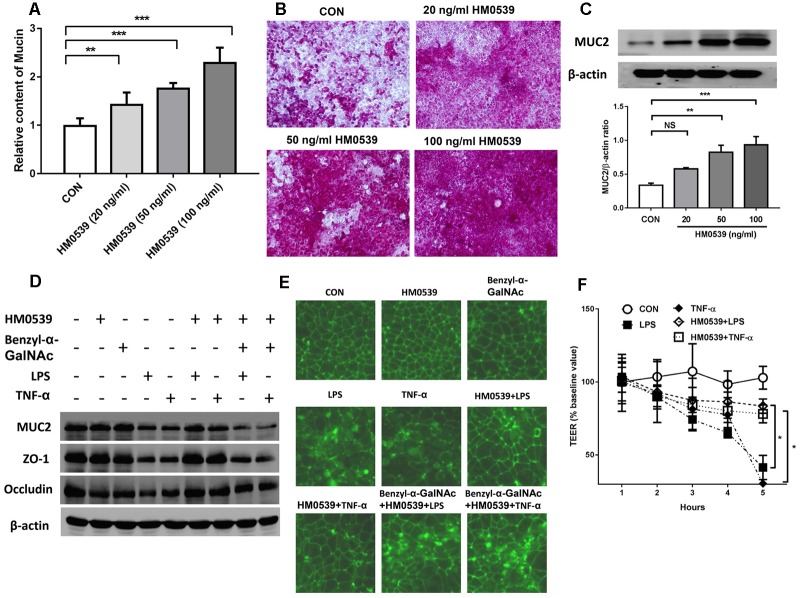
Protective effect of HM0539 on intestinal barrier function. **(A–C)** Caco-2 monolayers were pretreated with different concentrations of HM0539 (0–100 ng/ml), the soluble mucin **(A)** and mucin-covered Caco-2 surface **(B)** were detected by PAS assay. Western blot (**C**, upper panel) and densitometric analysis (**C**, lower panel) of MUC2 expression. **(D–F)** Caco-2 monolayers were pretreated with HM0539 or Benzyl-α-GalNAc (mucin inhibitor), then stimulated with LPS (1 μg/ml) or TNF-α (10 ng/ml) for 6 h. **(D)** Western blot analysis of MUC2, ZO-1 and occludin, β-actin was used as loading control. **(E)** Immunofluorescent staining of ZO-1. **(F)** TEER were detected to evaluate the intestinal barrier integrity. Data are given as means ± SEM; ^∗^*P* < 0.05, ^∗∗^*P* < 0.01, ^∗∗∗^*P* < 0.001; NS, no statistical significance.

We next evaluated the effects of HM0539 on intestinal mucin production *in vitro*. Caco-2 monolayers were incubated with different concentrations of HM0539 (0–100 ng/ml), and production of mucin in soluble ingredients or covered the Caco-2 monolayers was detected. We found that HM0539 could significantly induce mucin production and MUC2 expression in a dose-dependent manner (Figure 2A–C), implying that HM0539 has the same bioactivity as LCS in its mucin-enhancing ability. Then, we investigated whether HM0539 could protect the intestinal barrier from LPS- or TNF-α-induced injury. Western blot analysis showed that stimulation with LPS or TNF-α significantly reduced the expression of intestinal TJ protein (ZO-1, occludin) and intestinal mucin (MUC2) (Figure 2D). However, pretreatment of Caco-2 monolayer with HM059 could obviously abolish LPS- or TNF-α-induced downregulation of these proteins. Interestingly, when using Benzyl-α-GalNAc, a potent inhibitor of mucin, the protective effect of HM0539 on ZO-1 expression was inhibited, indicating that HM0539 may protect ZO-1 partially via enhancing intestinal mucin production (Figure 2D). Further, immunofluorescence staining of ZO-1 showed that control, HM0539 and Benzyl-α-GalNAc-treated Caco-2 monolayer display a typical chicken wire pattern of ZO-1 staining. However, this typical morphology was broken when Caco-2 was stimulated with LPS or TNF-α (Figure 2E). In contrast, pretreatment of Caco-2 with HM0539 restores the typical morphology of ZO-1 (Figure 2E). Finally, we noted that LPS or TNF-α stimulation decreases the TEER of Caco-2 monolayer, which is a useful index for evaluating the intestinal barrier integrity. Similarly, pretreatment of Caco-2 with HM0539 suppress LPS- or TNF-α-induced TEER decline, inferring that HM0539 can maintain the intestinal integrity against LPS- and TNF-α-induced disruption (Figure 2F). Together, these results suggested that HM0539 could protect intestinal barrier function from LPS- or TNF-α-induced injury.

### Treatment With HM0539 Reduces the Susceptibility of Neonatal Rat to *E. coli* K1 Infection via the Oral Route

**FIGURE 3:**
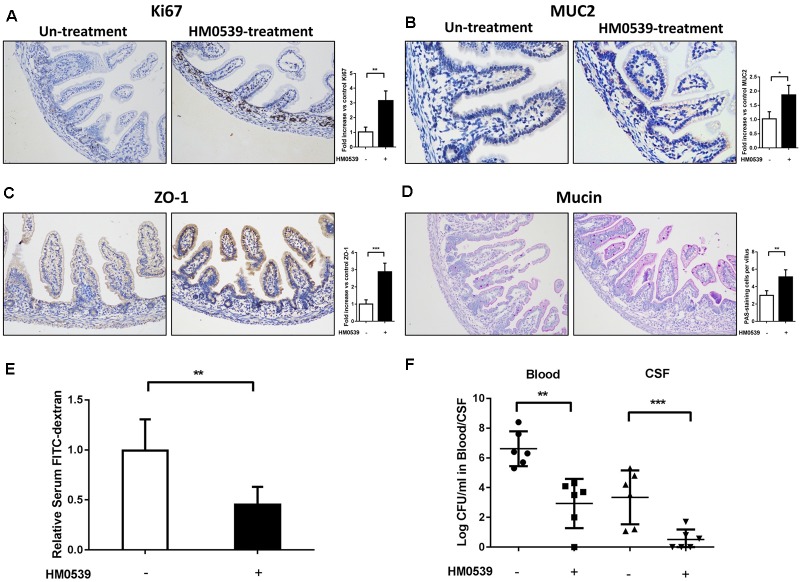
Effect of HM0539 on maturation of neonatal rat intestinal defense and gut-derived *E. coli* K1-induced infection. **(A–D)** Neonatal rats were orally administrated with pectin/zein control or pectin/zein beads containing HM0539 for 3 days, then the colon tissue was extracted for evaluation of intestinal defense. Immunohistochemical staining (left panel) and semiquantitative analysis (right panel) of Ki67 **(A)**, MUC2 **(B)**, and ZO-1 **(C)** of the colon section. **(D)** PAS staining of the colon section for evaluating the production of mucin, right panel showed the PAS stain-positive cells per villus. **(E,F)** Neonatal rats were treated as described above and were gavage fed with 5 × 10^9^ CFU *E. coli* K1, blood and CSF were harvested 60 h later. Intestinal permeability was evaluated by FITC-dextran assay. **(E)** Relative FITC-dextran level in serum. **(F)** Bacterial counts in blood and CSF. Data are given as means ± SEM; ^∗^*P* < 0.05, ^∗∗^*P* < 0.01, ^∗∗∗^*P* < 0.001.

Because HM0539 has the same mucin-enhancing ability as LCS *in vitro*, we then determined whether HM0539 has potential to protect neonatal rats from *E. coli* K1 infection via the oral route. We first explored the role of HM0539 on maturation of neonatal intestinal defense as described previously (He et al., 2017). Newborn rats were gavaged with pectin/zein control or pectin/zein beads containing HM0539 once a day for 3 days without *E. coli* K1 infection. Then, colon tissues of all rats were extracted and used for immunohistochemical analysis of the expressions of Ki67, MUC2, ZO-1, and mucin. We found that administration with HM0539 could significantly enhance expression of these molecules (Figure 3A–D), implying that HM0539 has the potential to accelerate the maturation of neonatal intestinal defense as did LCS. Moreover, the FITC-dextran assay showed that administration of HM0539 could reduce the intestinal permeability of the neonatal rat (Figure 3E). At last, we determined whether HM0539 could enhance the resistance of neonatal rat to *E. coli* K1, which is a common intestinal bacteria caused neonatal sepsis and meningitis via intestinal translocation. Newborn rats were gavaged with pectin/zein control or pectin/zein beads containing HM0539 once a day for 3 days, followed by intragastric administration of 5 × 10^9^ CFU *E. coli* K1. As expected, administration of HM0539 confers a higher resistance for neonatal rats against oral *E. coli* K1 infection, as reflected by lower *E. coli* K1 counts in blood and CFS from HM0539-treated mice than those from the control group (Figure 3F). Collectively, these results indicated that HM0539 is sufficient to protect neonatal rats against *E. coli* K1 pathogenesis.

### Treatment With HM0539 Protects Mice Against DSS-Induced Colitis

**FIGURE 4:**
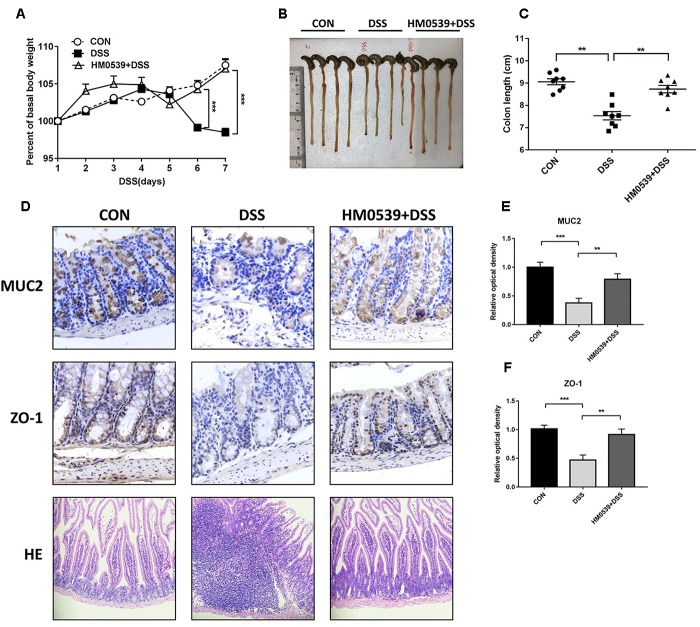
Protective effect of HM0539 on DSS-induced colitis. C57BL/6 mice were randomly divided into control group (CON), DSS-colitis group (DSS) and HM0539-treated DSS-colitis group (HM0539 + DSS). Mice were gavage fed with pectin/zein control or pectin/zein beads containing HM0539 from days 1 to 9. DSS colitis was induced by adding 3% DSS in the drinking water from days 3 to 9. All mice were sacrificed at day 12, the colitis severity and intestinal dysfunction were evaluated. **(A)** Body weight of mice from 1 to 7 days after DSS treatment. **(B,C)** Colon length of mice at day 12. Immunohistochemical staining and semiquantitative analysis of MUC2 (**D**, upper panel, **E**) and ZO-1 (**D**, middle panel, **F**) of the colon section. (**D**, lower panel) HE staining of colon section. Data are given as means ± SEM; ^∗∗^*P* < 0.01, ^∗∗∗^*P* < 0.001.

Given that HM0539 has the potential to exert a beneficial effect on the intestinal barrier function, we next extended its role to prevent the common intestinal barrier dysfunction-associated disease, including colitis and liver disease-associated bacterial translocation and liver injury. Colitis is a typical intestinal integrity disruption disease (Choksi et al., 2018). Here, we used the DSS-induced colitis model, a well-established model of acute intestinal mucosal injury, to further investigate the intestinal protective effect of HM0539. Mice were treated with pectin/zein control or pectin/zein beads containing HM0539 as described in the Section “Materials and Methods.” Colitis was induced by drinking 3% DSS water. Body weight, colon length, intestinal injury and expression of MUC2 and ZO-1 were recorded, detected and analyzed for evaluation of colitis severity. We found DSS-colitis mice suffered from more body weight loss after 5–7 days of DSS treatment than HM0539 + DSS-colitis mice (Figure 4A). Simultaneously, colon length of DSS-colitis mice was also significantly reduced compared to those of HM0539 + DSS-colitis mice (Figure 4B,C). In agreement with these results, we observed that DSS-colitis mice displayed marked histopathological changes in HE-stained colon, with more severe crypt loss and leukocyte infiltration than in HM0539 + DSS-colitis mice (Figure 4D, lower panel). Consistent with the pathological changes in the colon, we found that HM0539 treatment restores DSS-induced reduction of intestinal ZO-1 and MUC2 levels [Figure 4D (upper and middle panel), E,F]. Together, these results demonstrated that HM0539 has a preventive effect against DSS-induced colitis.

### Treatment With HM0539 Prevents LPS/GalN-Induced Intestinal Barrier Dysfunction, Bacterial Translocation and Liver Injury

**FIGURE 5:**
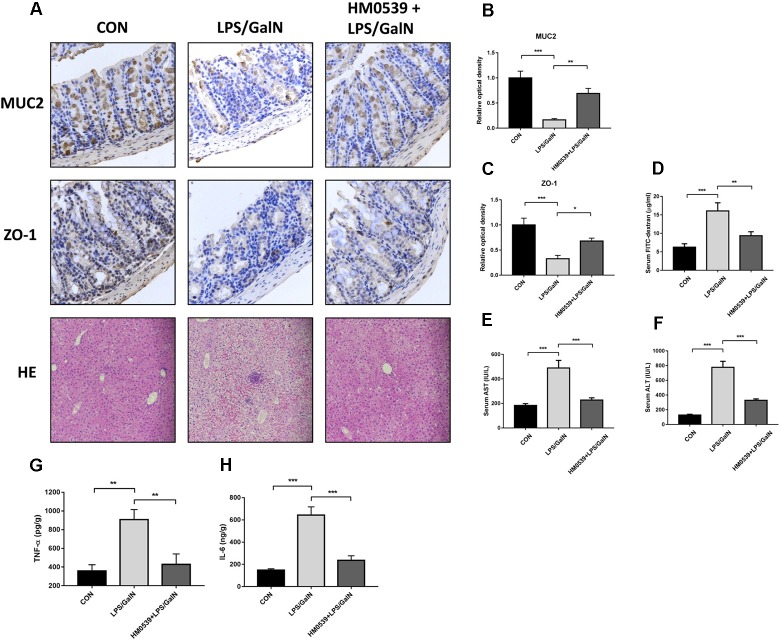
Protective effect of HM0539 on LPS-GalN-induced intestinal barrier dysfunction, bacterial translocation and liver injury. C57BL/6 mice were randomly allocated into control group (CON), LPS/GalN group (LPS/GalN), and HM0539-treated LPS/GalN group (HM0539 + LPS/GalN). Mice were gavage fed with pectin/zein control or pectin/zein beads containing HM0539 for 7 days. Then, mice were intraperitoneally injected with saline or LPS and GalN. All mice were sacrificed 24 h later. Bacterial translocation, intestinal barrier dysfunction and liver injury were evaluated. Immunohistochemical staining and semiquantitative analysis of MUC2 (**A**, upper panel, **B**) and ZO-1 (**A**, middle panel, **C**) of the colon section. Intestinal permeability was evaluated using FITC-dextran assay. (**A**, lower panel) HE staining of liver section. **(D)** Relative FITC-dextran level in serum. **(E,F)** AST and ALT analysis of hepatic homogenate. **(G,H)** ELISA analysis of TNF-α and IL-6 in hepatic homogenate. Data are given as means ± SEM; ^∗^*P* < 0.05, ^∗∗^*P* < 0.01, ^∗∗∗^*P* < 0.001.

Next, we determined whether HM0539 could exert a protective effect against LPS/GalN-induced intestinal barrier dysfunction, bacterial translocation and liver injury. C57BL/6 mice were intragastrically administrated with pectin/zein control or pectin/zein beads containing HM0539 for 7 days, followed by intraperitoneally injected with LPS and GalN. Colon and liver tissues were extracted 24 h later for immunohistochemical analysis and HE staining, respectively. We found that stimulation of mice with LPS/GalN reduced the intestinal expression of MUC2 and ZO-1 [Figure 5A (upper and middle panels), B,C], as well as enhanced the intestinal permeability (Figure 5D). In contrast, administration with HM0539 restored these parameters. Correspondingly, bacterial translocation to liver and MNL was also considerably inhibited in the HM0539-treated group when compared with the LPS/GalN group (Table 3). These data indicated that HM0539 could prevent against LPS/GalN-induced intestinal barrier dysfunction and bacteria translocation.

**Table 3:** Bacterial counts in liver and MLN homogenate.

Group	Affected/Total Number		Range (CFU/g of tissue)	
	
	Liver	MLN	Liver	MLN
Control (*n* = 7)	0/7	0/7	0	0
LPS/GalN (*n* = 7)	6/7	7/7	827.6 ± 1015	573.4 ± 735.8
HM0539 + LPS/ GalN (*n* = 7)	2/7	2/7	57.43 ± 104.8 (^∗^)	11 ± 20.81 (^∗^)


*^∗^P < 0.05 compared with LPS/GalN group*.

Finally, we investigated whether HM0539 could prevent LPS/GalN-induced hepatic damage. HE staining of liver section showed that LPS/GalN induces significant liver injury compared with the control group (Figure 5A lower panel), including hemorrhaging, microabscesses and extensive macrophage and neutrophil infiltration. In contrast, HM0539-treated mice exhibited a normal histology. Moreover, the serum AST and ALT levels decreased significantly in the HM0539 + LPS/GalN group when compared with those in the LPS/GalN group, confirming that HM0539 could prevent LPS/DalN-induced liver injury (Figure 5E,F). Moreover, we noted that HM0539 treatment attenuated LPS/DalN-induced secretion of proinflammatory cytokine TNF-α and IL-6 in hepatic homogenate (Figure 5G,H), indicating that HM0539 also exerts an anti-inflammatory property in the liver injury context. Altogether, these data suggest that HM0539 is useful in preventing LPS/GalN-induced intestinal barrier dysfunction, bacteria translocation and liver injury.

## Discussion

Postbiotics have recently received medical attention for their potential beneficial effects on host health. Here we identified and characterized a novel postbiotic, named HM0539, from the culture supernatant of LGG. We found this postbiotic could protect intestinal epithelium from LPS- or TNF-α-induced injury. Further *in vivo* studies demonstrated that HM0539 could not only prevent DSS-induced colitis but also LPS/GalN-caused intestinal barrier dysfunction, bacteria translocation and liver injury. Our research provides new insight into the mechanism of probiotic action, and a novel idea for prevention and treatment of intestinal barrier dysfunction-associated diseases.

*Escherichia coli* equipped with K1 capsule has the potential to cross the intestinal barrier and the blood–brain barrier to cause neonatal sepsis and meningitis (Coureuil et al., 2017). The intestinal colonization and translocation of *E. coli* K1 are critical for its bloodstream dissemination and eventual systemic infection and meningitis (Croxen and Finlay, 2010; He et al., 2017; Zeng et al., 2017). Neonates, especially preterm infants and those with very low birth weights, are susceptible to *E. coli* K1 infection due to their immature intestinal barrier defense (Croxen and Finlay, 2010; Birchenough et al., 2013; Birchenough et al., 2017; He et al., 2017; Zeng et al., 2017). In our previous study, it was shown that treatment with LCS enhances the resistance of neonatal rats to oral *E. coli* K1 infection via promoting the maturation of neonatal intestinal defense. However, the exact component of LCS which exerts this barrier enhancement and disease-protective effect remained unknown. In this study, we found HM0539, a protein secreted from LCS identified by LC-MS/MS, was sufficient to accelerate the maturation of neonatal intestinal defense and protect them from *E. coli* K1 infection via oral route. These data lead us to conclude that HM0539 is the bioactive constituent of LGG culture supernatant, and also support the theory of introducing probiotic-derived products to replace live probiotics to avoid the potential risks in certain conditions. For special populations, such as preterm infants, those with low birth weight, immunocompromised patients etc., administration of probiotics must be done very carefully, because many probiotics, including LGG, were reported to induce bacteremia and sepsis (Brecht et al., 2016; Dani et al., 2016). In our study, the finding that probiotic-derived protein has the potential to exert a potent protective effect provides new ideas for maintaining health in special populations.

An interesting finding here is that we did not find p40 from LGG culture supernatant. In fact, p40 and p75 were the two most abundant proteins purified from LGG culture supernatant previously by Yan et al. (2007). Subsequent studies showed that p40 can inhibit cytokine-induced intestinal epithelial apoptosis, enhances intestinal mucin and IgA production and preserves intestinal barrier function (Yan et al., 2007, 2013; Yan and Polk, 2012; Wang et al., 2014, 2017). Although both p40 and p75 have potential to modulate intestinal homeostasis, p40 exerts more potent effects than p75 (Yan and Polk, 2012). Here, we identified at least 58 proteins from LCS, among which, however, HM0539, but neither p40 nor p75, was the most abundant one. This result is consistent with a previous study reported by Sánchez et al. (2009), in which there is also no trace of p40. Surprisingly, in that study, the most abundant protein is p75 but not HM0539. These inconsistent results among Yan, Sánchez and our own group may be due to the different procedures in preparing LGG culture supernatant, because different culture conditions may affect the secretion protein of LGG (Koskenniemi et al., 2009). Although it is different from p40 and p75, HM0539 also has potential to protect the intestinal barrier. We thus conclude that HM0539 is a novel LGG postbiotic. Studies are in progress to compare the function between p40 and HM0539.

Since HM0539 has a distinct role in intestinal barrier protection, we next explore its therapeutic potential by employing two disease models typically related to intestinal barrier dysfunction. The first model we employed was DSS-induced colitis, which is one of the most commonly used animal models of colitis for developing and evaluating potential therapeutic strategies (Kraus and Arber, 2009). The mechanism by which DSS induces colonic mucosal injury remains unclear, but recent studies demonstrated that sulfate groups of the DSS molecules disrupt the intestinal mucus layer and make the intestinal barrier more permeable to antigens (Eichele and Kharbanda, 2017). In fact, the detrimental effect of DSS on the intestinal barrier function was detectable already after 4–12 h of stimulation. However, at this point, there is no significant clinical symptom of colitis (Petersson et al., 2010). Furthermore, Sharma et al. (2018) showed that loss of epithelial integrity precedes overt intestinal inflammation and injury. These studies demonstrated that failing intestinal barrier function may play as a cause of colitis, thus protection of intestinal integrity by HM0539 could improve the overall disease.

The second model we employed was LPS-GalN-induced acute liver failure. Although there is a lot of evidence that GalN induces liver injury directly, studies have demonstrated that bacteria and its products such as endotoxin, are a pivotal determinant for the pathogenesis of this liver injury (Ewaschuk et al., 2007). The intestinal integrity is disrupted in liver failure, leading to translocation of bacteria and its products to the systemic circulation. It has been clearly shown that GalN-induced gut-derived bacteria/endotoxin translocation induces Kupffer cell activation and release of TNF-α, eventually resulting in severe hepatic impairment (Morita et al., 2004). Furthermore, it was found that monoclonal antibodies to endotoxin and removal of bacteria from the gut (such as antibiotics treatment or colectomy) both block GalN-induced liver injury (Morita et al., 2004). Thus, like the DSS-colitis model, intestinal barrier dysfunction is also a critical trigger of inflammation in the LPS/GalN model. This scenario may explain why HM0539 could relieve liver injury in this liver failure model. Taken together, these findings suggested that HM0539 has not only great potential in preventing intestinal barrier dysfunction, but also an effective prophylaxis for gut barrier dysfunction-derived diseases. Further study is necessary to elucidate the beneficial mechanism of HM0539.

A limitation that should be addressed here is the incomprehensive understanding of the beneficial role of HM0539 on intestinal barrier function. As discussed above, HM0539 achieved its disease-protective effects mainly through enhancement of the intestinal physical barrier function, such as the mucus layer, TJs expression and intestinal integrity. However, the gut immune system and gut microbiota are also critical constituents of the intestinal barrier function (Maloy and Powrie, 2011; Deshmukh et al., 2014; Lv et al., 2014). In fact, the gut microbiota, gut immune system and gut physical barrier have a complex interaction with each other in order to maintain the intestinal homeostasis. Disruption or alteration of one of them can lead to changing in the other aspects (Peterson and Artis, 2014). For example, mucus can form a special structure to maintain the high concentrations of antimicrobial molecules (defensins and secretory IgA) close to the intestine epithelial layer, which is crucial for maintaining the gut microbiota (Maloy and Powrie, 2011). In turn, the microbiota can also affect intestinal MUC2 mucin *O*-glycosylation (Arike et al., 2017). The mucus and mucins of the goblet cells and enterocytes can interact with and modulate the gut immune system (Pelaseyed et al., 2014). Intestinal epithelial cells, the main body of the intestinal physical barrier, are always functioning as a mediator of intestinal homeostasis, through releasing of factors that influence microbial colonization, sensing of both beneficial and harmful microbes, and regulating intestinal immune responses (Maloy and Powrie, 2011). So the relationship among intestinal physical barrier, gut immune system and gut microbiome is mutually beneficial. Given that HM0539 exerts a potent beneficial effect on intestinal physical barrier, we believe it also has great potential in the modulation of the gut immune system and gut microbiota.

In summary, we identified and purified a novel LGG-soluble protein named HM0539 and characterized its intestinal barrier protective function with respect to promoting mucin secretion and TJ protein expression and reducing gut permeability. Further, we explored its therapeutic potential by introducing two barrier dysfunction associated models: DSS-induced colitis and LPS-GalN-induced acute liver failure. These results support the potential wide application of HM0539 in diseases related to gut barrier dysfunction, while the exact mechanism needs further investigation.

## Author Contributions

HC, S-HH, JG, XH, YL, and YuW conceived and designed the experiments. JG, YL, YuW, TH, LL, ZG, QZ, SY, YiW, WY, and ZZ performed the experiments. JG, ZG, LL, WY, SY, YuW, and HC analyzed the data. S-HH contributed reagents, materials, and analysis tools. JG, HC, YuW, YL, and TH participated in its design and coordination and helped to draft the manuscript. All authors read and approved the final manuscript.

## Conflict of Interest Statement

The authors declare that the research was conducted in the absence of any commercial or financial relationships that could be construed as a potential conflict of interest.

## Footnotes

1 http://www.ncbi.nlm.nih.gov/Genbank/index.html

## Supplementary Material

FIGURE S1 Bioinformatic analysis and purification of HM0539. **(A)** Amino acid sequence alignments between HM0539, hydrolase and previously identified p40 and p75. Sequences were identified using a BLAST search and aligned using DNAMAN program. **(B)** The presence and location of signal peptide cleavage sites in HM0539 were predicted using SignalP 4.1 Server. **(C)** Prediction of transmembrane helice(s) of HM0539 were performed using TMHMM Server 2.0. **(D)** HM0539 was recombined and purified using the His-tag/Ni-NTA system. Lane 2: cell lysates; Lanes: 3–5: eluted HM0539.

FIGURE S2 Genetic map of loci required for the secretion of HM0539. The exact location of HM0539 in the genetic locus was shown by a solid black arrow. The locations and directions of the transcription of the genes are represented by arrows, and the corresponding protein names are shown above or below them. A scale (in kilobases) is shown at the bottom.

FIGURE S3 Amino acid alignment of HM0539 and its homologous protein from *Lactobacillus rhamnosus* strain ATCC 21052, *Lactobacillus rhamnosus* strain NCTC13764, *Lactobacillus rhamnosus* strain LR-B1, *Lactobacillus rhamnosus* strain LR5, *Lactobacillus rhamnosus* HN001, *Lactobacillus casei* strain LC5, *Lactobacillus casei* ATCC 393.

TABLE S1 Full-length coding DNA of HM0539 protein.

TABLE S2 Amino acid sequences comparison results of 19 best matched homologous proteins to HM0539.
