# Supplementary figures and images for: A Novel Postbiotic From Lactobacillus rhamnosus GG With a Beneficial Effect on Intestinal Barrier Function
Source: Front Microbiol. 2019 Mar 14;10:477. doi: 10.3389/fmicb.2019.00477 (PMC6426789; doi:10.3389/fmicb.2019.00477)

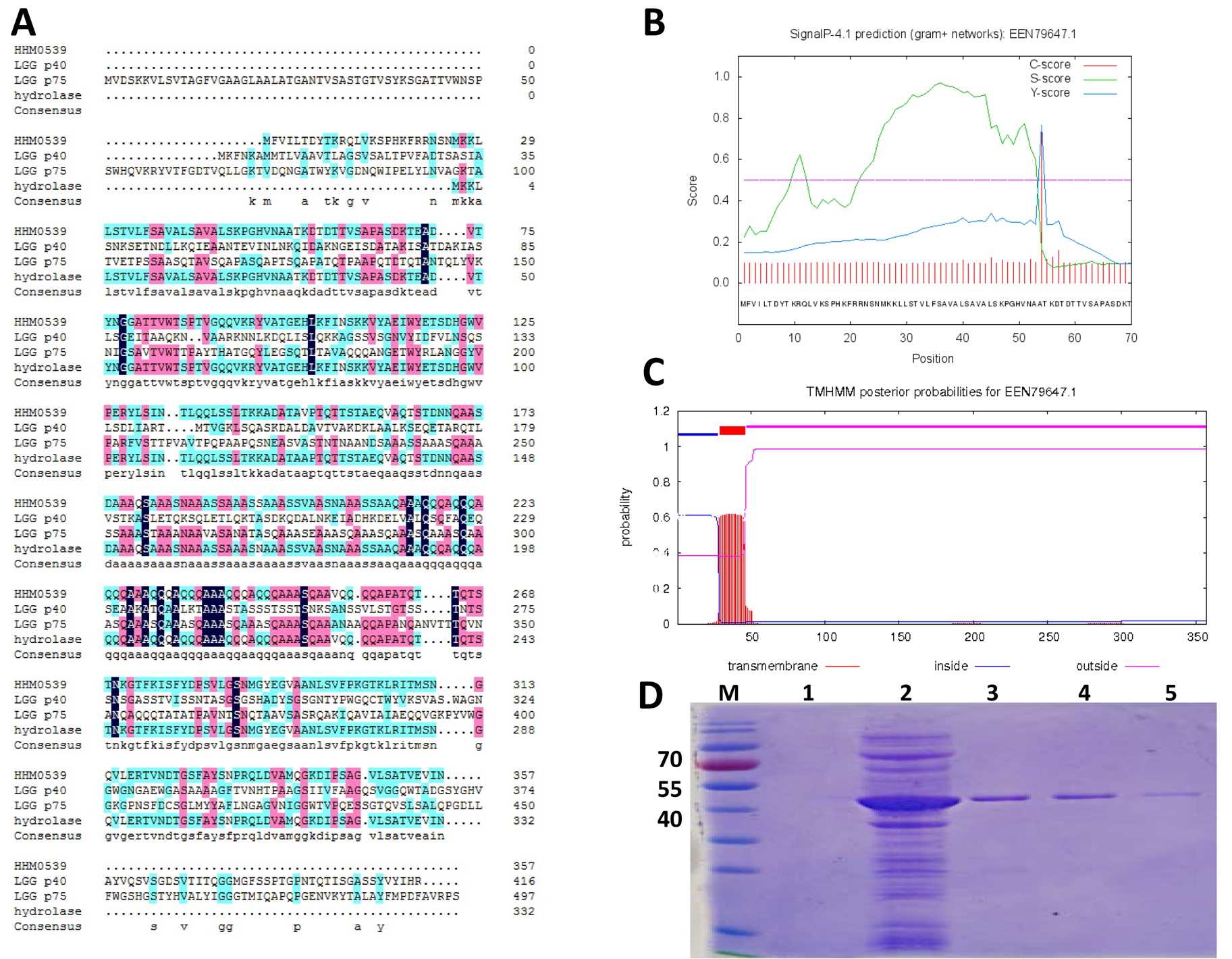

Supplement: FIGURE S1 — Bioinformatic analysis and purification of HM0539. (A) Amino acid sequence alignments between HM0539, hydrolase and previously identified p40 and p75. Sequences were identified using a BLAST search and aligned using DNAMAN program. (B) The presence and location of signal peptide cleavage sites in HM0539 were predicted using SignalP 4.1 Server. (C) Prediction of transmembrane helice(s) of HM0539 were performed using TMHMM Server 2.0. (D) HM0539 was recombined and purified using the His-tag/Ni-NTA system. Lane 2: cell lysates; Lanes: 3–5: eluted HM0539. [file Image_1.JPEG]

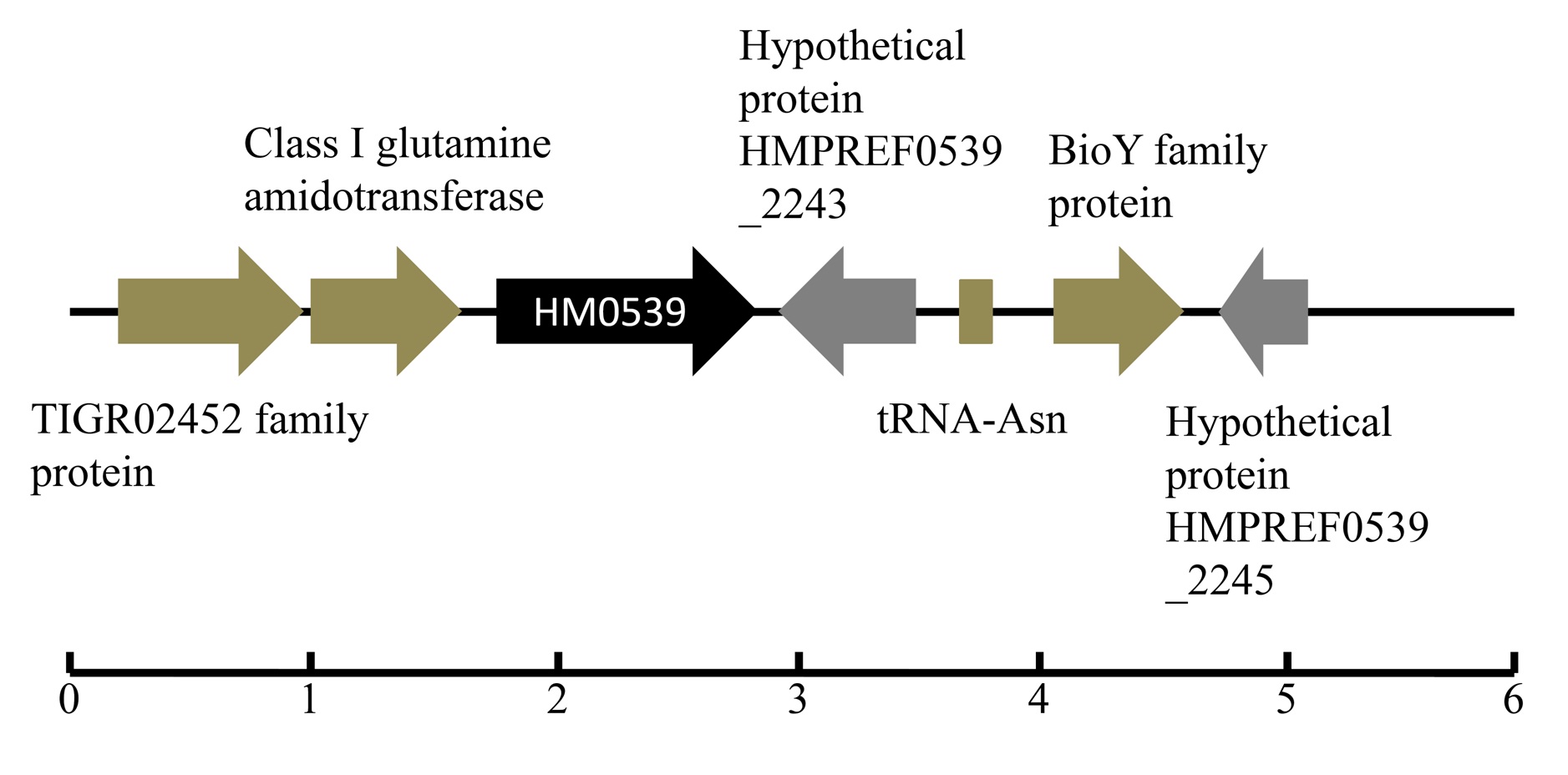

Supplement: FIGURE S2 — Genetic map of loci required for the secretion of HM0539. The exact location of HM0539 in the genetic locus was shown by a solid black arrow. The locations and directions of the transcription of the genes are represented by arrows, and the corresponding protein names are shown above or below them. A scale (in kilobases) is shown at the bottom. [file Image_2.JPEG]

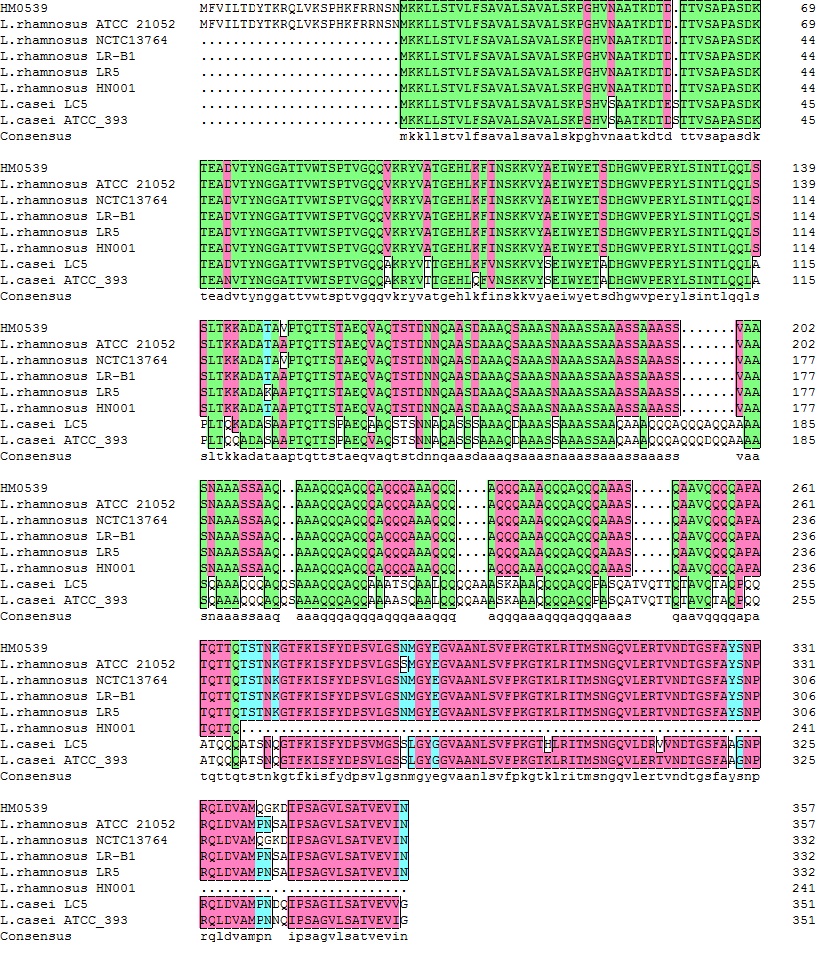

Supplement: FIGURE S3 — Amino acid alignment of HM0539 and its homologous protein from Lactobacillus rhamnosus strain ATCC 21052, Lactobacillus rhamnosus strain NCTC13764, Lactobacillus rhamnosus strain LR-B1, Lactobacillus rhamnosus strain LR5, Lactobacillus rhamnosus HN001, Lactobacillus casei strain LC5, Lactobacillus casei ATCC 393. [file Image_3.JPEG]
