# Supplementary material for: A Novel Postbiotic From Lactobacillus rhamnosus GG With a Beneficial Effect on Intestinal Barrier Function
Source: Front Microbiol. 2019 Mar 14;10:477. doi: 10.3389/fmicb.2019.00477 (PMC6426789; doi:10.3389/fmicb.2019.00477)
Supplement: TABLE S1 — Full-length coding DNA of HM0539 protein. [file Table_1.docx]

**Supplementary Table 1. Full-length coding DNA of HM0539 protein.**

| **Full-length coding DNA of HM0539** |
| --- |
| \| d \| ATGTTCGTAA \| TACTCACTGA \| CTATACTAAG \| CGACAGTTAG \| TTAAGTCACC \| \| --- \| --- \| --- \| --- \| --- \| --- \| \| 51 \| ACACAAATTC \| AGGAGGAACT \| CGAACATGAA \| AAAGCTCTTA \| AGTACAGTAT \| \| 101 \| TATTCTCAGC \| CGTTGCTTTA \| TCTGCCGTTG \| CCTTGTCAAA \| ACCAGGTCAC \| \| 151 \| GTTAACGCGG \| CAACGAAAGA \| TACTGACACC \| ACCGTCAGCG \| CACCAGCCAG \| \| 201 \| GTTAACGCGG \| CAACGAAAGA \| TACTGACACC \| ACCGTCAGCG \| CACCAGCCAG \| \| 251 \| TGATAAGACT \| GAAGCTGATG \| TCACTTATAA \| TGGCGGTGCA \| ACAACCGTTT \| \| 301 \| GGACCTCACC \| GACTGTGGGA \| CAGCAAGTTA \| AGCGTTATGT \| AGCAACCGGT \| \| 351 \| GAACATCTTA \| AATTTATTAA \| TAGTAAAAAA \| GTTTACGCAG \| AAATCTGGTA \| \| 401 \| CGAAACATCT \| GACCATGGTT \| GGGTTCCAGA \| ACGTTATTTG \| AGCATCAACA \| \| 451 \| CCTTGCAGCA \| ATTATCATCA \| TTGACCAAGA \| AGGCTGATGC \| CACTGCTGTG \| \| 501 \| CCAACTCAGA \| CAACCTCGAC \| TGCGGAACAG \| GTTGCGCAAA \| CATCCACAGA \| \| 551 \| TAATAATCAG \| GCTGCATCCG \| ATGCTGCTGC \| TCAAAGTGCT \| GCAGCTTCTA \| \| 601 \| ATGCGGCTGC \| CTCAAGCGCC \| GCTGCATCAA \| GCGCTGCAGC \| TTCCAGCGTT \| \| 651 \| GCGGCATCCA \| ACGCTGCTGC \| TTCCAGCGCG \| GCTCAGGCTG \| CGGCTCAACA \| \| 701 \| GCAGGCGCAA \| CAACAAGCTC \| AGCAACAGGC \| TGCGGCTCAA \| CAACAGGCAC \| \| 751 \| AACAGCAAGC \| CGCTGCTCAG \| CAACAAGCAC \| AGCAGCAGGC \| AGCTGCATCT \| \| 801 \| CAGGCTGCCG \| TTCAACAACA \| GCAAGCACCA \| GCAACCCAGA \| CGACCCAAAC \| \| 851 \| ATCAACTAAT \| AAGGGCACTT \| TTAAAATTAG \| TTTCTATGAC \| CCGTCTGTTT \| \| 901 \| TAGGTAGCAA \| CATGGGTTAC \| GAAGGTGTGG \| CTGCAAACCT \| GAGCGTCTTC \| \| 951 \| CCTAAAGGTA \| CTAAGCTGCG \| AATCACCATG \| TCTAACGGAC \| AAGTGTTAGA \| \| 1001 \| ACGAACCGTT \| AATGATACTG \| GCTCATTTGC \| ATATAGCAAC \| CCGCGCCAGC \| \| 1051 \| TTGACGTCGC \| AATGCAGGGT \| AAGGACATTC \| CATCAGCCGG \| AGTGCTTTCT \| \| 1101 \| GCAACCGTTG \| AAGTGATCAA \| CTAA \|  \|  \| |

**Supplementary Table 2.** Amino acid sequences comparation results of 19 best matched homologous proteins to HM0539.

| **Protein** | **Strains** | **Length** | **Percent similarity** | **Percent identity** |
| --- | --- | --- | --- | --- |
| Hypothetical protein | *L. rhamnosus* strain ATCC 21052 | 357 | 98.6 % | 98.3 % |
| Cell wall-associated hydrolase | *L. rhamnosus* strain NCTC13764 | 332 | 93.0 % | 93.0 % |
| Cell wall-associated hydrolase | *L. rhamnosus* strain NCTC13710 | 332 | 93.0 % | 93.0 % |
| Hydrolase | *L. rhamnosus* strain ASCC 290 | 332 | 93.0 % | 93.0 % |
| TolA protein | *L. rhamnosus* strain LOCK908 | 332 | 93.0 % | 93.0 % |
| Cell wall-associated hydrolase | *L. rhamnosus* strain ATCC 8530 | 332 | 93.0 % | 93.0 % |
| Putative protein without homology | *L. rhamnosus* strain Lc 705 | 332 | 93.0 % | 93.0 % |
| Hydrolase | *L. rhamnosus* strain LR-B1 | 332 | 91.8 % | 91.6 % |
| Hydrolase | *L. rhamnosus* strain 4B15 | 332 | 91.8 % | 91.6 % |
| Hydrolase | *L. rhamnosus* strain WQ2 | 332 | 91.8 % | 91.6 % |
| Hydrolase | *L. rhamnosus* strain BFE5264 | 332 | 91.8 % | 91.6 % |
| Hydrolase | [*L. rhamnosus* strain LRB](https://blast.ncbi.nlm.nih.gov/Blast.cgi#alnHdr_1062772459) | 332 | 91.8 % | 91.6 % |
| Hydrolase | [*L. rhamnosus* strain LR5](https://blast.ncbi.nlm.nih.gov/Blast.cgi#alnHdr_1239728832) | 332 | 91.6 % | 91.3 % |
| Hypothetical protein N507_0959 | [*L. rhamnosus* strain DSM 14870](https://blast.ncbi.nlm.nih.gov/Blast.cgi#alnHdr_1240584818) | 332 | 91.6 % | 91.3 % |
| Hydrolase | [*L. rhamnosus* strain Pen](https://blast.ncbi.nlm.nih.gov/Blast.cgi#alnHdr_1172545480) | 332 | 91.6 % | 91.3 % |
| TolA protein | [*L. rhamnosus* strain LOCK900](https://blast.ncbi.nlm.nih.gov/Blast.cgi#alnHdr_521374308) | 332 | 91.6 % | 91.3 % |
| Cell wall-associated hydrolase | [*L. rhamnosus* strain HN001](https://blast.ncbi.nlm.nih.gov/Blast.cgi#alnHdr_183393127) | 241 | 67.5 % | 67.2 % |
| Hydrolase | *L.casei* strain LC5 | 351 | 73.7 % | 70.5 % |
| Hydrolase | [*L. casei* strain ATCC 393](https://blast.ncbi.nlm.nih.gov/Blast.cgi#alnHdr_530674796) | 351 | 73.7 % | 71.6 % |
